# Biomimicry of quorum sensing using bacterial lifecycle model

**DOI:** 10.1186/1471-2105-14-S8-S8

**Published:** 2013-05-09

**Authors:** Ben Niu, Hong Wang, Qiqi Duan, Li Li

**Affiliations:** 1 College of Management, Shenzhen University, Shenzhen 518060, China; 2 Department of Industrial & Systems Engineering, The Hong Kong Polytechnic University, Hong Kong; 3 Hefei Institute of Intelligent Machines, Chinese Academy of Sciences, Hefei 230031, China

## Abstract

**Background:**

Recent microbiologic studies have shown that quorum sensing mechanisms, which serve as one of the fundamental requirements for bacterial survival, exist widely in bacterial intra- and inter-species cell-cell communication. Many simulation models, inspired by the social behavior of natural organisms, are presented to provide new approaches for solving realistic optimization problems. Most of these simulation models follow population-based modelling approaches, where all the individuals are updated according to the same rules. Therefore, it is difficult to maintain the diversity of the population.

**Results:**

In this paper, we present a computational model termed LCM-QS, which simulates the bacterial quorum-sensing (QS) mechanism using an individual-based modelling approach under the framework of Agent-Environment-Rule (AER) scheme, i.e. bacterial lifecycle model (LCM). LCM-QS model can be classified into three main sub-models: chemotaxis with QS sub-model, reproduction and elimination sub-model and migration sub-model. The proposed model is used to not only imitate the bacterial evolution process at the single-cell level, but also concentrate on the study of bacterial macroscopic behaviour. Comparative experiments under four different scenarios have been conducted in an artificial *3-D *environment with nutrients and noxious distribution. Detailed study on bacterial chemotatic processes with quorum sensing and without quorum sensing are compared. By using quorum sensing mechanisms, artificial bacteria working together can find the nutrient concentration (or global optimum) quickly in the artificial environment.

**Conclusions:**

Biomimicry of quorum sensing mechanisms using the lifecycle model allows the artificial bacteria endowed with the communication abilities, which are essential to obtain more valuable information to guide their search cooperatively towards the preferred nutrient concentrations. It can also provide an inspiration for designing new swarm intelligence optimization algorithms, which can be used for solving the real-world problems.

## Background

Many agent-based models, inspired by biological phenomenon, have been formulated to provide new approaches for solving realistic optimization problems especially complex NP problems [1]. Two different types of agent-based models, population-based and individual-based models, have been classified based on the viewpoint of biological simulation. In population-based models such as the particle swarm optimization algorithm [2], all individuals have unique characters and follow the same evolutionary rules. Nevertheless, in individual-based models (IBM) [3], an individual is regarded as a discrete entity endowed with its own attributes, states and behaviors. Every heterogeneous entity can communicate with each other and then make group decisions by social intelligence.

Early in 1988, Kreft and his colleagues proposed an individual-based model termed *BacSim *to simulate the evolution process of Escherichia coli (*E. coli*) from an individual bacterium to a group. As he says, we can see a macroscopic world in the microscopic object [4]. An *E-CELL *model was illustrated by Tomita et al. in 1999, inspired by developmental processes of Mycoplasma genitalium [5]. Ginovart et al. (2002) designed a discrete *IBM *called *INDISIM *to simulate the growth of bacterial cultures [6]. An alternative model based on the *COSMIC *system to simulate the artificial bacterial interaction and evolution was shown by Paton et al. in 2004 [7]. Soon after, Emonet et al. (2005) developed an IBM termed *AgentCell *to simulate bacterial chemotactic processes at the single-cell level [8]. Another individual-based model of low-population bacteria cultures in the lag stage was presented by Prats et al. in 2006 [9]. Recently, an IBM termed *iDynoMiCS*, which employs new bacterial biofilm modelling approaches, was formulated by Lardon et al. (2011) [10].

In our previous work, we formulated a lifecycle model (LCM) guided by the Agent-Environment-Rule architecture to simulate the bacterial evolution in 2008 [11]. LCM mainly focuses on microscopic and macroscopic evolution processes of bacteria in different growth phases. Three main developmental phases of *E. coli *including the lag, dynamic and decline phases are studied. Compare with the population-based computational model, the individual-based LCM has improved flexibility where the behaviors of every individual could be investigated and controlled. The original LCM, however, is in its infancy [11]. Recent studies have demonstrated that quorum sensing (QS) systems generally exist in bacteria acting as communication with and between groups [12]. Incorporating intra- and inter-species QS mechanisms into LCM is the primary aim and work of this paper.

## Methods

### Lifecycle model (LCM)

A bio-inspired lifecycle model (LCM), according to bacterial evolution processes during their lifecyle, was proposed as a new inspiration to solve optimization problems in 2008 [11]. Behaviors of *E. coli *in different life phases are concentrated on in LCM. In biological science, behaviors of *E. coli *have been intensively studied for more than 150 years and four key behavioral patterns of *E. coli*, i.e. *chemotaxis, reproduction, migration and elimination*, have been detailed described in [13].

In absence of gradient information about attractant or repellent chemical concentration, a bacterium runs in a straight line using flagella as propellers for a few seconds, and then tumbles with random directions. The run-tumble-run cycle will be repeated during the whole bacterial lifecycle. A bacterium with gradient information shows distinctly different behaviors. It has been suggested that the bacteria can possess the memory ability so that it can compare current gradient information with previous ones [14]. If the concentration of attractant chemicals raises or the density of repellent chemicals reduces, the frequency of run increases. Otherwise, the frequency of tumble increases. The run-tumble-run cycle is the essential property of bacterial chemotactic behaviors.

When a bacterium obtains sufficient energy from the environment, it has a chance to reproduce. The healthy bacterium splits into two identical daughter cells in the same spatial position. Those with poor nutrients intake may undergo extinction or migrate to new niches for survival [15]. Of course, the population varies between different life phases. In the lagging phase, most bacteria absorb rich nutrients and thus the population of bacteria grows exponentially. The increasing of population size leads to the intensified competition for nutrients. During dynamic phase, the population size of bacterial colony fluctuates markedly in the early stage and then gradually stagnates in a relatively stable state. With the food depleted gradually, some bacteria are not able to find sufficient food sources, which will be eliminated or migrate to new places with good nutrient concentrations, the population size reduces at the decline stage.

**Figure 1:**
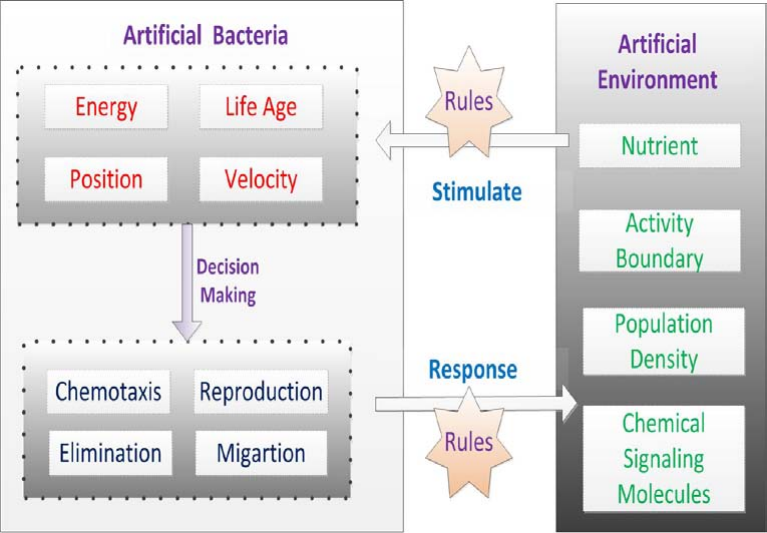
**Lifecycle model under the agent-environment-rule architecture**.

In LCM, each bacterium has different characters and is independent from each other. Therefore, it is also considered as the artificial individual possessing an ability of autonomous. LCM consists of three underlying components. The most important component is the artificial bacteria, which possesses plenty of attributes and behavioral features [16]. *N*-dimension environment with gradient information where artificial bacteria undertake metabolisms is the second key component. The most complex component is interaction rules between artificial bacteria and environment. The original LCM model is presented in Figure 1.

Although the theoretical modelling based on individual bacterial behaviors is insufficient. The flexible structure of LCM allows it to retain the potential of incorporating quorum-sensing (or cell-cell communication) mechanisms. This paper chiefly concentrates on the integration of QS systems into LCM for achieving a more accurate biological simulation model. A brief description of QS mechanisms is given as follows.

### Quorum-sensing (QS) mechanisms

New microbial discoveries have illustrated that though as the simple unicellular microbes on earth, bacteria can utilize cell-to-cell communication to make group decisions, synthesize beneficial molecules for themselves and so on [17]. Information about other bacteria and the environment can be acquired by an individual bacterium, and interpreted in a 'meaningful' way which finally results in sharing of knowledge [18]. Possessing the sophisticated linguistic communication abilities, bacteria are able to take on some advanced features of social intelligence, such as cooperative foraging and creating complex niches [19]. Such communication process via chemical signals is termed quorum sensing (QS).

**Figure 2:**
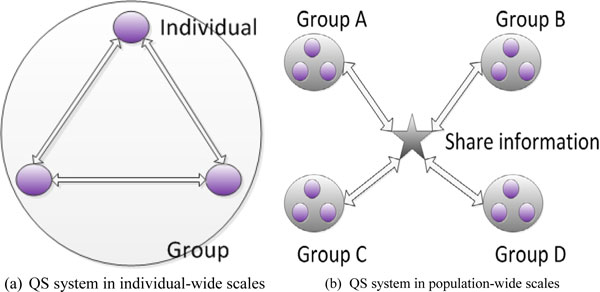
**Two kinds of topologic schemas of QS**.

We now know that QS systems have been found in both Gram-positive and Gram-negative bacteria [20]. For example, the first known QS mechanism was discovered in bioluminescent bacteria called *Vibrio fischeri*. It resides in light-producing organs of the squid for reproduction. *AI-1 *synthase produced by *Vibrio fischeri *diffuses *AHL *molecules to increase the cell-population concentration. As long as the cell-population density exceeds a threshold level, luciferase operons will be activated and result in the generation of light [21]. More and more studies have shown that the *AI-1 *QS system is mainly responsible for local interactions between colony members [22]. In contrast, another famous *AI-2 *QS system functions as interspecies communication [23]. According to different QS mechanisms, two kinds of topologic schemas are presented in Figure 2.

Two main kinds of QS systems in individual-wide and population-wide scales are considered and simulated in the paper [24]. It is clear that via the use of intrinsic QS mechanisms, global behaviors of bacterial species are coordinated for maximizing group benefits as well as individual benefits [25].

### LCM with QS mechanisms

**Figure 3:**
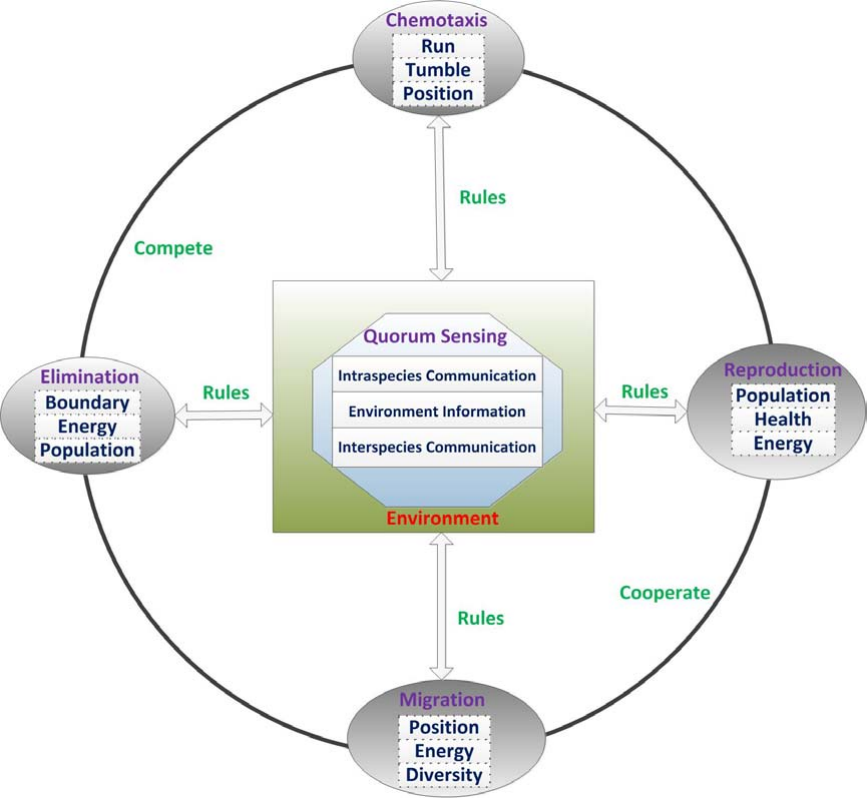
**LCM with quorum-sensing systems (LCM-QS)**.

Lifecycle model with QS mechanisms (LCM-QS) not only involves microscopic objects such as the run-tumble-run cycle, but also includes macroscopic entities such as the interspecies communication. LCM-QS is more in accordance with the natural metabolic processes compared with the original LCM. LCM-QS is made up of five core components, i.e., *chemotaxis, quorum sensing, reproduction, elimination and migration*, as shown in Figure 3.

The bacterial movement of chemotaxis is run through in the whole lifecyle. During each movement of run or tumble, each bacterium pursues nutrients or avoid noxious. Bacteria with high energy intake will broadcast their search information to other bacteria with low energy level by QS mechanism. When a mass of bacteria congregate together and the local environment becomes overcrowded, they compete with each other instead of cooperated with others. Some bacteria with strong foraging abilities accumulate sufficient nutrients for reproduction. Others that lack competitive edges are easily eliminated. In the proposed model, these dead bacteria are replaced by copies of bacteria possessing the opportunity of reproduction. The rest of bacteria, which have a little energy and average foraging capacities, will migrate to a new region together through interspecies communication. To reduce computational complexity, the lifecycle model with QS mechanisms is divided into three sub-models, which are presented in detail as follows.

### Chemotaxis with QS sub-model

**Figure 4:**
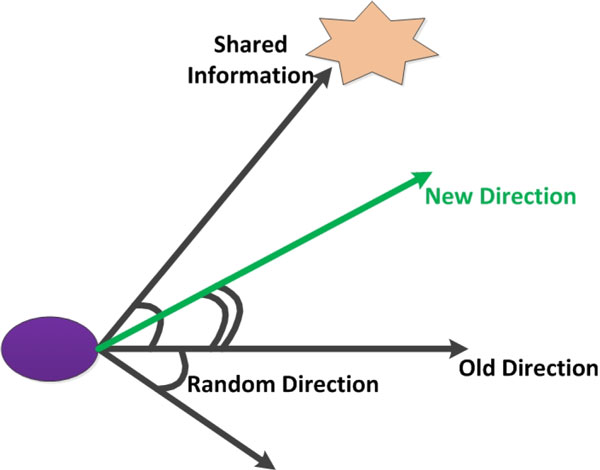
**Chemotactic behaviors with an intra-species QS mechanism**.

In the primary LCM, bacterial movement of runs and tumbles with no information exchange within and between bacterial strains, which is not in accord with recent biological discoveries. In fact, the chemotactic behavior is always accompanied with intra-species and inter-species communication via QS systems in the whole bacterial optimization processes. Microbial chemotactic behaviors are mainly influenced by personal previous experiences, information exchange and random direction choices, which are shown in Figure 4. The chemotactic behavior with QS mechanisms prolongs motion towards a favorable orientation and restrains movement in an adverse direction [26].

The specific formulae about bacterial motion are presented below. Equation 1 indicates chemotactic behaviors of simulated bacteria in the absence of QS mechanisms. On the contrary, artificial bacterial movement with QS systems is characterized in Equation 2. $P_{i}\left(k\right)$ donates position of the *i-*th bacterium in the *k-*th iteration step. *R *indicates a random number. $C_{i}$ is the run length of *i*-th bacterium. randDir(i,k) stands for a random orientation choice of the *i-*th bacterium in the *k-*th iteration step. Gbest donates current optimal location with richest nutrients.

(1) $$P_{i}\left(k+1\right)=P_{i}\left(k\right)+R^{*}{C_{i}}^{*}randDir\left(i,k\right)$$

(2) $$P_{i}\left(k+1\right)=P_{i}\left(k\right)+{R_{1}}^{*}{C_{i}}^{*}randDir\left(i,k\right)+{R_{2}}^{*}\left(Gbest-P_{i}\left(k\right)\right)$$

It is expected that the integration of QS mechanisms into chemotaxis will facilitate the cooperative search of the global optimum and accelerate the convergence rate. Subsequently, the reproduction and elimination sub-model will been conducted until the number of run and tumble reaches a certain value ($N_{re}$ or $N_{eli}$). Note that the number of chemotaxis step equals the total number of iterations (iterMax).

### Reproduction and elimination sub-model

After long-time chemotactic steps, some bacteria with higher energy level (represented by nutrient concentrations) have more opportunities to reproduce and maximize lifespan, whereas other bacteria with lower energy level are faced with being eliminated. Bacteria accumulate enough nutrients can propagate by binary fission and produce two identified daughter cells at the same position. Besides, if an artificial bacterium move out of the restricted area, it should be deleted and replaced by a new bacterium for better control of the model. Detailed conditions of reproduction or elimination are presented below in Equations 3 and 4.

(3) $$\text{if}\;J_{i}>J_{threshold}\;\&\&\;iter>N_{re},\;\text{then}\;i\in \;healthy\;\text{and}\;\text{reproduce}\;i$$

(4) $$\text{if}\;J_{i}<J_{threshold}\;\&\&\;iter>N_{eli},\;\text{then}\;i\in \;unhealthy\;\text{and}\;\text{eliminate}\;i$$

where $J_{i}$ is fitness value of *i*th bacterium, $J_{threshold}$ is a predefined threshold. The asexual reproduction of healthy bacteria doubles the population of the group. Nevertheless, the colony population size may shrink rapidly owing to the sudden death of a mass of unhealthy bacteria. Hence, the total number of artificial bacteria in the proposed model remains unchanged. From the viewpoint of computation, the reproduction and elimination progress may disturb chemotactic processes in the next iteration step. But more importantly, it could improve the computational speed and possibly find the global optimum.

### Migration sub-model

**Figure 5:**
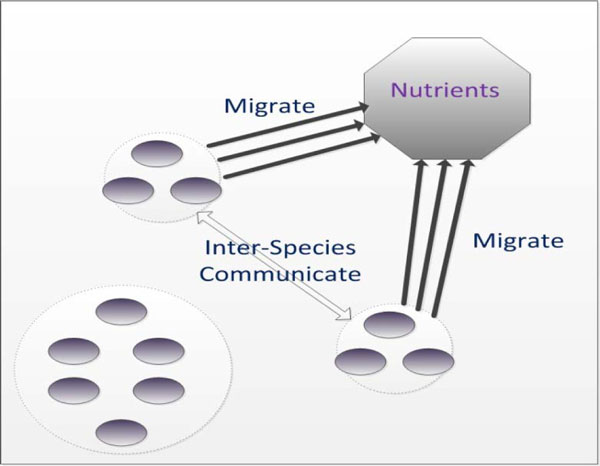
**Long-distance migration mechanism**.

Owing to the increment of bacteria in a given region, the competition for nutrients becomes more and more intensive. The nutrient-rich food sources will not satisfy the requirements of all bacteria. Naturally, some bacteria with average foraging capacities but poor energy level are more inclined to migrate to new areas with expected richer nutrient concentrations rather than die directly. From a perspective of optimization, the long-distance migration to new random niches, described in Figure 5, is able to keep the diversity of colony and avoid being trapped into local optimum.

A core formula of the migration sub-model is presented in Equation 5.

(5) $$P_{i}\left(d,k+1\right)=lb+rand^{*}{\left(d,k\right)}^{*}\left(ub-lb\right)$$

where lb indicates the lower boundary and ub donates the upper boundary, *d *is the number of the dimensions, *k *is the current chemotatic step. The values of lower and upper boundaries are always determined according to the constraints defined in the realistic optimization problems.

To simplify LCM-QS, it is suggested that the entire migration process will be conducted only if certain migration conditions are satisfied. For instance, if the chemotactic steps reach a predefined threshold value, the migration process will be performed.

### Implementation of LCM-QS

**Figure 6:**
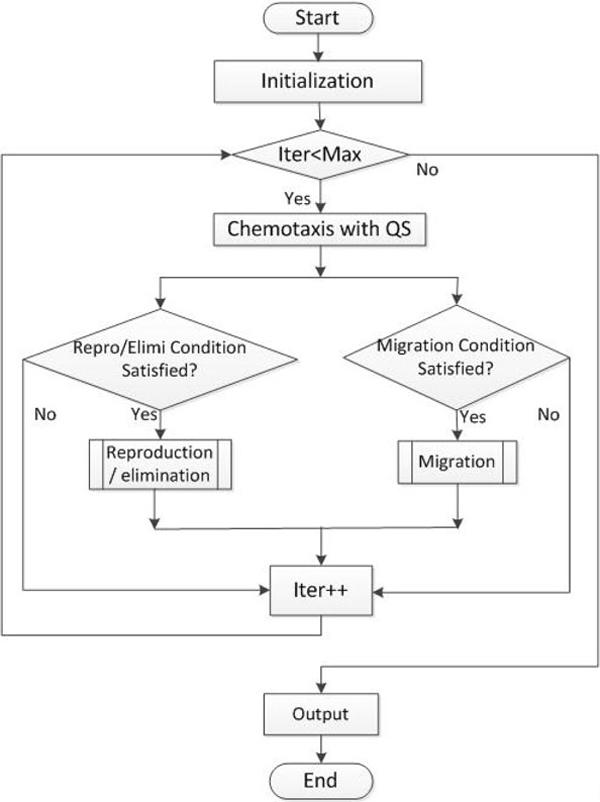
**Flow chart of simulation processes**.

Some population-based optimization models, such as BFO [13], utilize a nested loop structure, which requires more computational time and thus influences the convergence rate. However, LCM-QS adapts a sequential implementation structure to reduce computational time. In our proposed LCM-QS, a bridge between individual behaviors and group interactions is built and a right balance between computational simplicity and model complexity is maintained. The implementation procedure of LCM-QS is presented in Figure 6.

It should be noted that some initial parameters, such as boundary conditions and the bacterial initial position, are under user control. Table 1 gives a detailed description of the optimization processes of LCM-QS. It is obvious that the reproduction and elimination sub-model as well as the migration sub-model are condition-dependent.

**Table 1:** Implementation of LCM-QS

**For **(each loop or *iter*<*iterMax*)
// chemotaxis process
**For **(each bacterium )
**If **(given probability >random)
Share information with surrounding neighbours
Tumble ($P_{i}\left(k+1\right)=P_{i}\left(k\right)+R_{1}*C_{i}*randDir\left(i,k\right)+R_{2}*\left(Gbest-P_{i}\left(k\right)\right)$)
**While **(current function value *Fc*< previous function value *Fp*)
Swim (using Equation 2)
**End While**
**Else**
Share the information with two random-choice bacteria
Tumble (using Equation 2)
**While **(current function value *Fc*< previous function value *Fp*)
Swim (using Equation 2)
**End While**
**End if**
**End For**
// reproduction or elimination
**If **(reproduction conditions meet)
Sort and Split
Reproduction and elimination (using Equations 3 and 4)
**End If**
// migration
**For **(each bacterium)
**If **(a given probability *Nmig *> random)
Migrate to a new niche (using Equation 5)
**End if**
**End For**
**End For**

## Results and discussion

To measure the search performance of artificial bacteria using the proposed new model, simulation studies have conducted in a 3-D environment with nutrient-noxious distribution. As illustrated above, information exchange mechanism is one of key indicators in swarm optimization, our experimental studies are conducted with four types of information exchange scenarios.

A: Bacterial chemotaxis without information exchange;

B: Bacterial chemotaxis with group information exchange;

C: Bacterial chemotaxis with individual information exchange;

*D: Bacterial chemotaxis with individual and group information exchange*.

**Figure 7:**
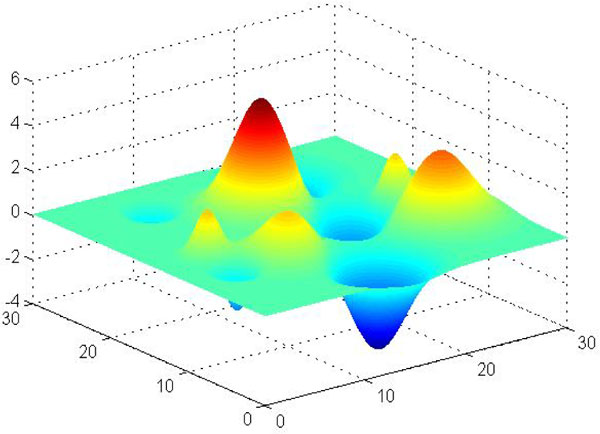
**Nutrient-noxious environments**.

The nutrient distribution of 3-D environment is set by the function as Equation (6), which is also illustrated in Figure 7.

(6) $$\begin{array}{llll} F\left(x\right)= & 5^{*}{\text{exp}}^{-0.1\left({\left(x_{1}-15\right)}^{2}+{\left(x_{2}-20\right)}^{2}\right)}-2^{*}{\text{exp}}^{-0.08\left({\left(x_{1}-20\right)}^{2}+{\left(x_{2}-15\right)}^{2}\right)}\; & & \;\\ & +3^{*}{\text{exp}}^{-0.08\left({\left(x_{1}-25\right)}^{2}+{\left(x_{2}-10\right)}^{2}\right)}+2^{*}{\text{exp}}^{-0.1\left({\left(x_{1}-10\right)}^{2}+{\left(x_{2}-10\right)}^{2}\right)}\; & & \;\\ & -2^{*}{\text{exp}}^{-0.5\left({\left(x_{1}-5\right)}^{2}+{\left(x_{2}-10\right)}^{2}\right)}-4^{*}{\text{exp}}^{-0.1\left({\left(x_{1}-15\right)}^{2}+{\left(x_{2}-5\right)}^{2}\right)}\; & & \;\\ & -2^{*}{\text{exp}}^{-0.5\left({\left(x_{1}-8\right)}^{2}+{\left(x_{2}-25\right)}^{2}\right)}-2^{*}{\text{exp}}^{-0.1\left({\left(x_{1}-21\right)}^{2}+{\left(x_{2}-25\right)}^{2}\right)}\; & & \;\\ & +2^{*}{\text{exp}}^{-0.5\left({\left(x_{1}-25\right)}^{2}+{\left(x_{2}-16\right)}^{2}\right)}+2^{*}{\text{exp}}^{-0.5\left({\left(x_{1}-5\right)}^{2}+{\left(x_{2}-14\right)}^{2}\right)}\; & & \;\\ & \; & \end{array}$$

### A: Bacterial chemotaxis without information exchange

**Figure 8:**
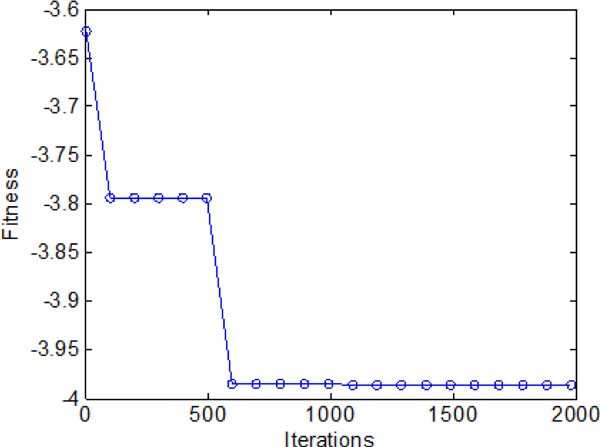
**The average fitness values during 2000 iterations without information exchange**.

**Figure 9:**
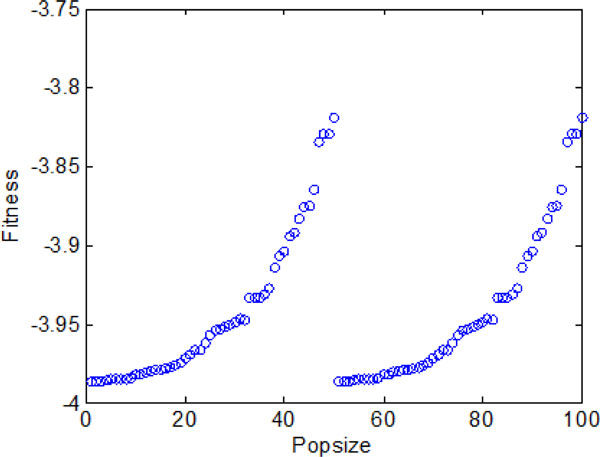
**The final optimal function values of each individual without information exchange**.

In this section, bacterial chemotaxis will be operated without considering information exchange between individuals and groups. Bacterium runs and tumbles to nutrition area by stochastic turbulence. Figure 8 shows the bacterial optimization process with the chemotaxis step $N_{c}$ ranging from 1 to 2000. In LCM-QS, chemotaxis goes along with entire optimization process. From Figure 8, the bacterial colonies have to spend more than 500 chemotaxis steps to find the global optimum. After a long time of chemotaxis (without communication), reproduction and elimination process, the final fitness value of each bacterium have been shown in Figure 9.

**Figure 10:**
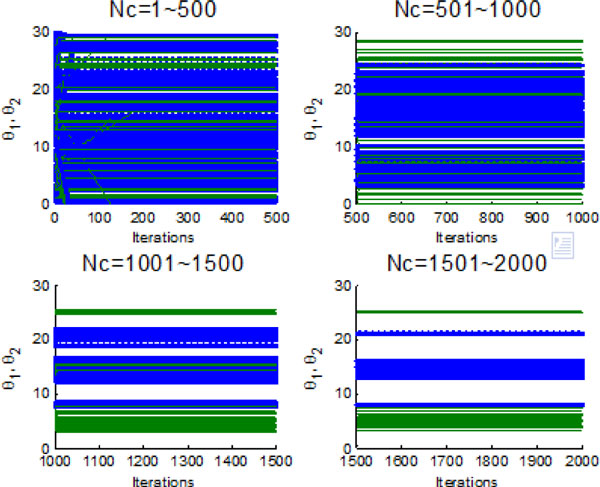
**2-D position during 2000 iteration process without information exchange**.

**Figure 11:**
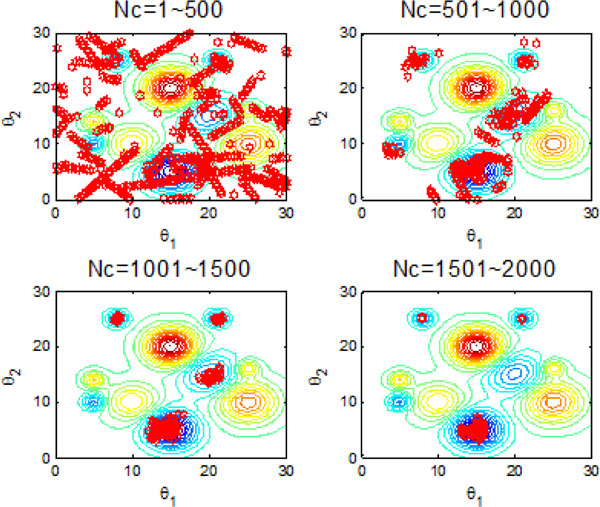
**The process of finding the global optimum without information exchange**.

Figure 10 and Figure 11 indicate the individual best position ($\theta_{1}$ and $\theta_{2}$ are two dimensional vectors) obtained by each bacterium during 2000 chemotactic step. Figure 10 points out that not all of bacteria in group find the global optimum after the maximal iterations satisfied, which is also confirmed by Figure 11. From Figure 11, chemotaxis process is divided into four stages and every stage has 500 chemotatic steps. But even in the fourth stage, some of bacteria cannot find the global optimum.

### B: Bacterial chemotaxis with group information exchange

**Figure 12:**
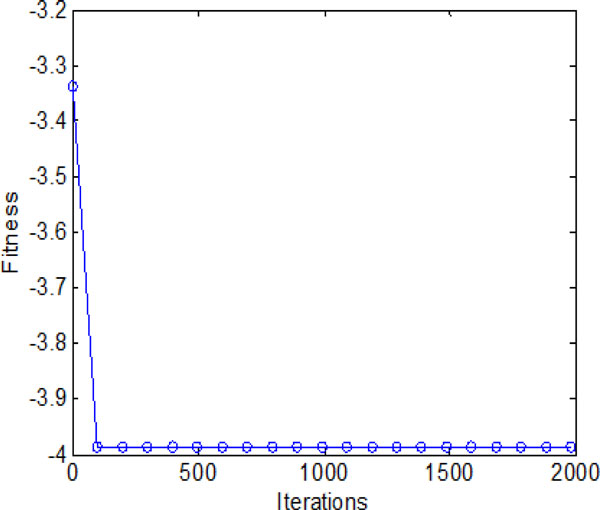
**The average fitness values during 2000 iterations with group information exchange**.

**Figure 13:**
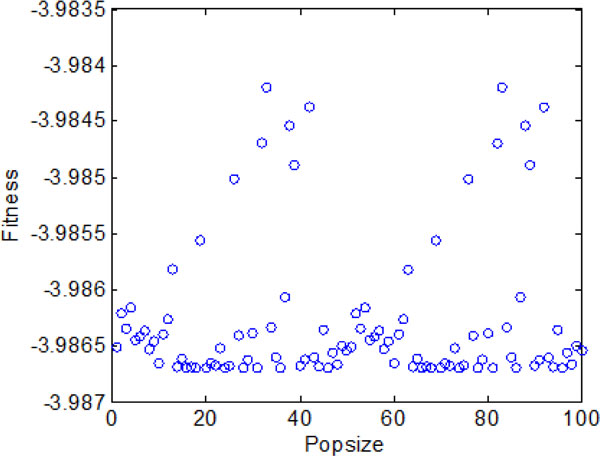
**The final optimal function values of each individual with group information exchange**.

**Figure 14:**
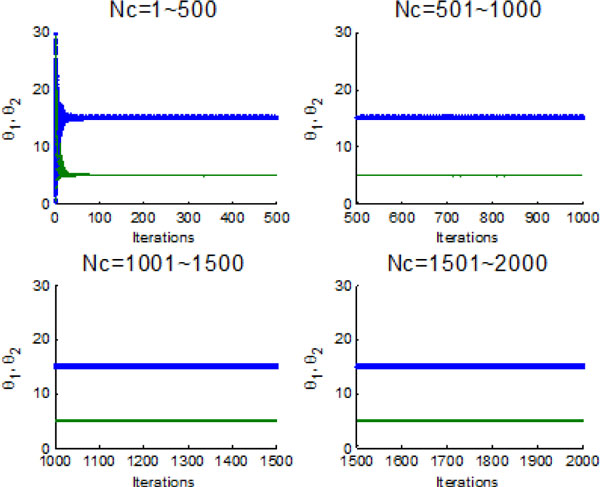
**2-D position during 2000 iteration process with group information exchange**.

**Figure 15:**
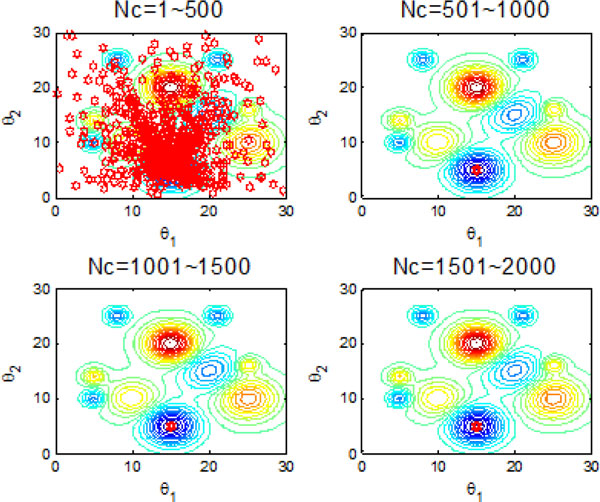
**The process of finding the global optimum with group information exchange**.

Section A suggests that the bacterial chemotaxis without information exchange fails to find global optimum efficiently. Therefore, bacterial chemotaxis with group information exchange is considered for getting better search performance in this section. Similar to the above section, Figure 12 shows the average optimal fitness obtained by bacterial colonies over 2000 chemotactic steps. The bacterial colonies approach the best nutrient concentrations with a faster convergence rate compared to the case of no information exchange. The individual best position found by each bacterium during 2000 chemotactic step is shown in Figure 14 and Figure 15. From the figures, we can find that most bacteria can reach the global optimum position in the first stage. Because most of the them can locate in the global optimum in the first stage, the best position obtained by bacterial colonies keep nearly unchanged at the later three stages. Specifically, in Figure 13, the fitness of the bacteria mostly assemble between -3.986 and -3.987 with little difference, and only a few of individuals cannot arrive at the global optimum but run very close to it.

### C: Bacterial chemotaxis with individual information exchange

**Figure 16:**
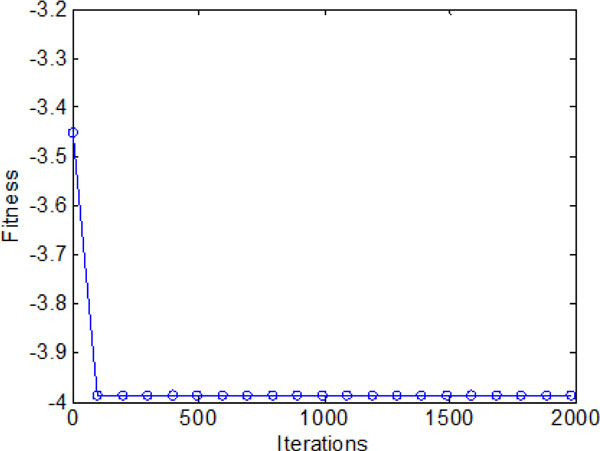
**The average fitness values during 2000 iterations with individual information exchange**.

**Figure 17:**
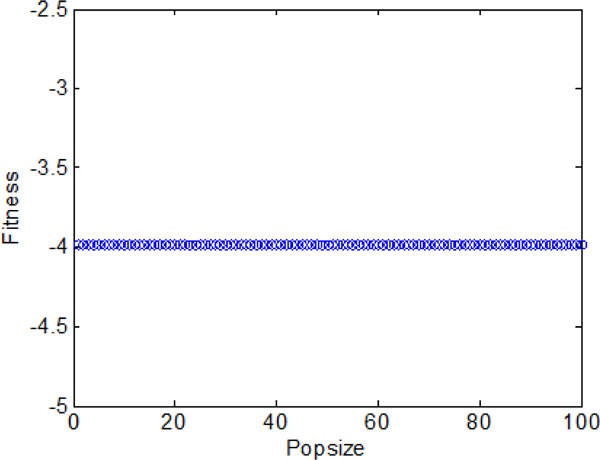
**The final optimal function values of each bacterium with individual information exchange**.

**Figure 18:**
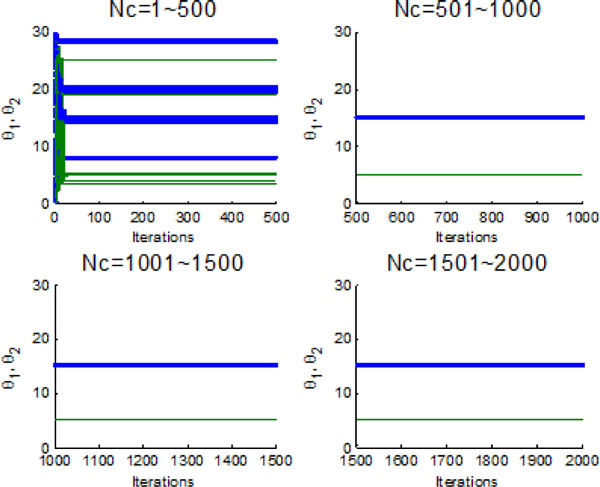
**2-D position during 2000 iteration process with individual information exchange**.

**Figure 19:**
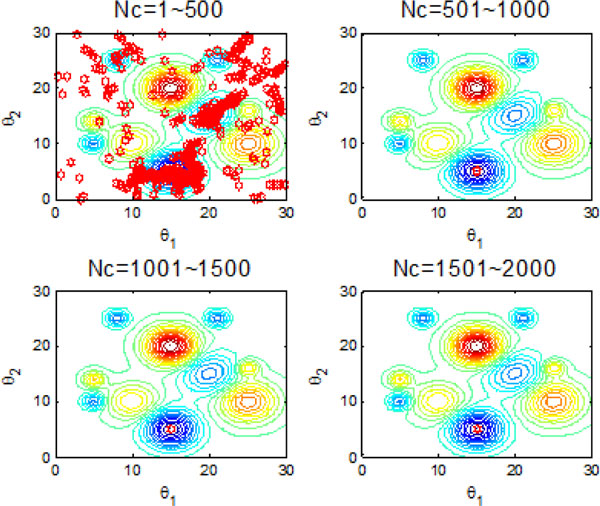
**The process of finding the global optimum with individual information exchange**.

Section B aims at improving the search efficiency of bacterial chemotaxis through group information exchange. In this section, individual information exchange is incorporated as one of communication mechanisms to speed up the search speed of global optimum. Figure 16 confirms that the participating of individual information exchange indeed helps a lot in guiding the bacteria to approach the nutrient area. After maximum iterations reached, all bacteria can find the global optimum shown in Figure 17. Figure 18 and Figure 19 once again illustrate that the individual communication mechanism is favorable to orient bacteria colony to global optimum. With the help of the individual information exchange between bacteria, the bacterial colony can find the global optimum in the first 500 chemotatic steps.

### D: Bacterial chemotaxis with individual and group information exchange

**Figure 20:**
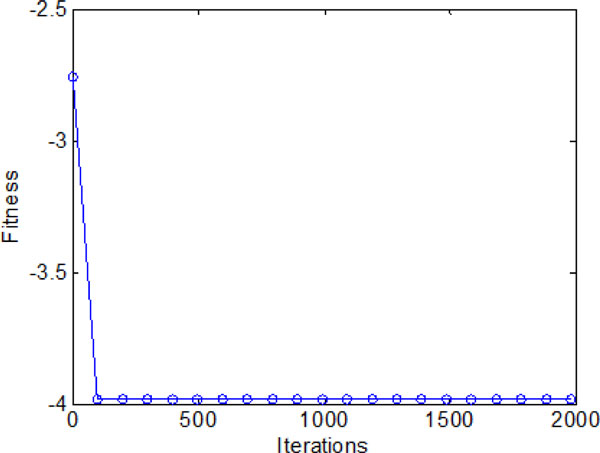
**The average fitness values during 2000 iterations with both individual and group information exchange**.

The average fitness values obtained by bacterial colony with individual and group information exchange are shown in Figure 20. It converges in a high speed in the early iterations (chemotatic step), but a relatively slow convergence rate in the later iterations. When approach the promising area (near global optimum), many iterations are used to fine-tune the local search. Finally, all the bacteria are able to find the global optimum when the maximum iterations reached.

**Figure 22:**
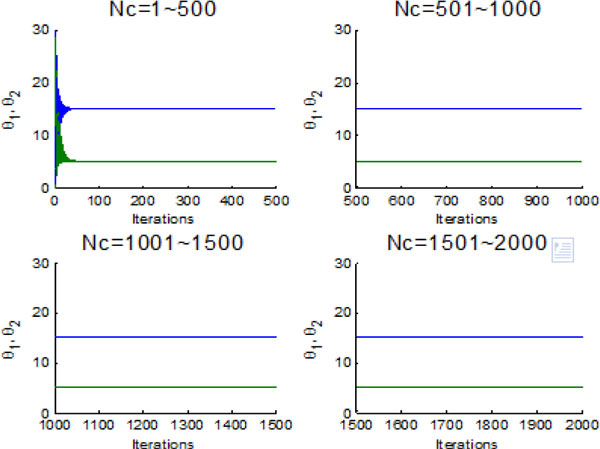
**2-D position during 2000 iteration process with both individual and group information exchange**.

**Figure 23:**
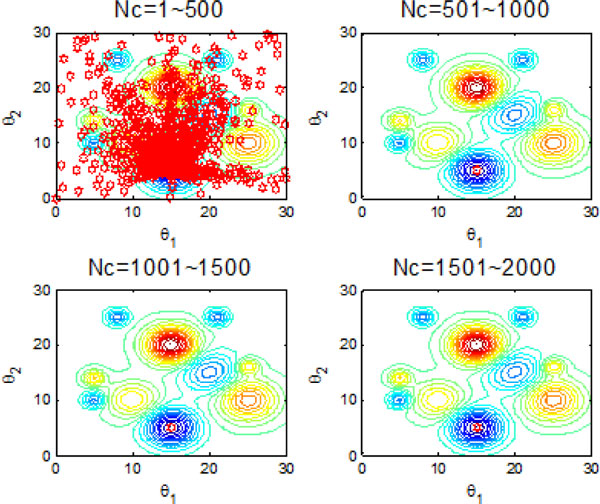
**The process of finding the global optimum with both individual and group information exchange**.

Figure 22 and Figure 23 inform that that bacteria can search for global optimum quickly with help of individual and group information exchange. Figure 22 even shows that the bacteria can find the global optimum no more than 100 iterations. There is no doubt that these two communication mechanisms have increasingly improved the search efficiency of original model with no information exchange or individual information exchange.

**Figure 24:**
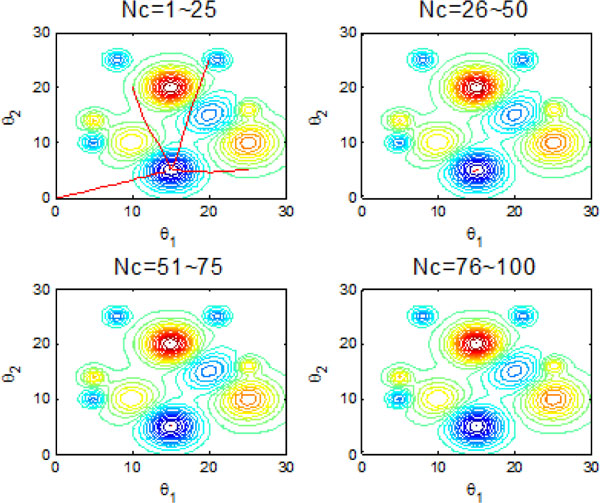
**Global optimum found by four bacteria during 100 chemotactic steps**.

**Figure 25:**
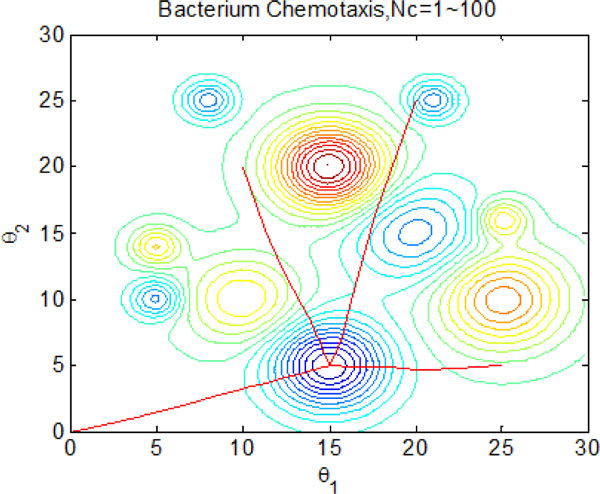
**Optimal process of four bacteria during 100 chemotactic steps**.

**Figure 26:**
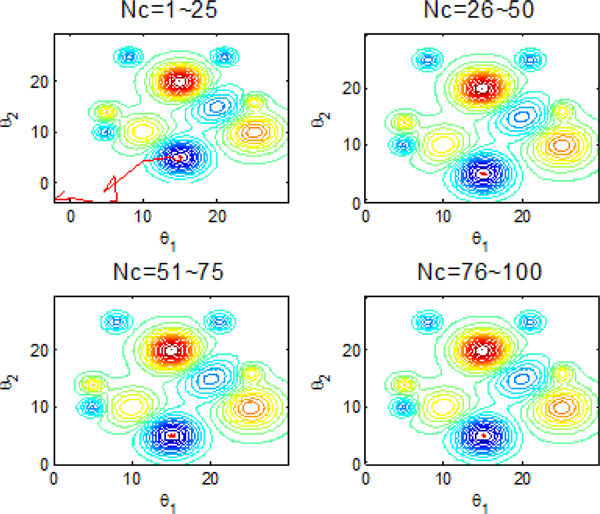
**Global optimum found by single bacterium during 100 chemotactic steps**.

**Figure 27:**
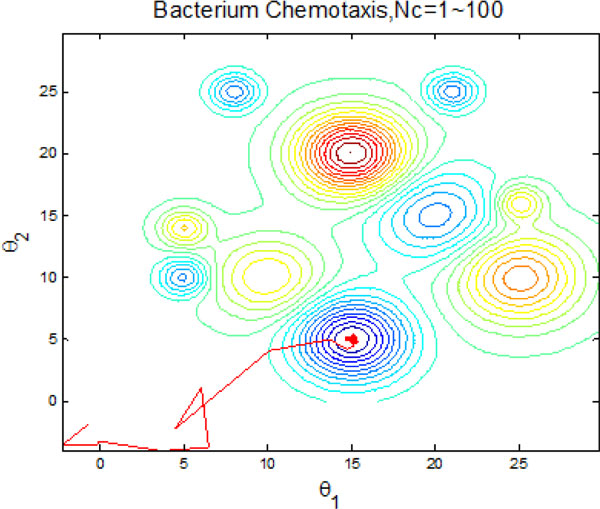
**Optimal process of one bacterium during 100 chemotactic steps**.

The results presented in the above figures indicate the emergent behavior of comparative search from a macroscopic view. LCM-QS model is in relating the macroscopic effects of bacterial colony to microscopic behavior of single bacterial cell. Figure 24 and Figure 25 describe optimization process in bacterial micro-communities, where four bacteria are selected. Those four bacteria are randomly distributed in the artificial environment. After 100 iterations, all of them located the global optimum with the best nutrient concentrations. Chemotaxis process of a single bacterium during four different stages (25 chemotatic steps in one stage) is shown in Figure 26. From the figure we can find that the bacterium located in the global optimum position with the best nutrient concentrations after 25 steps. The overall search procedure during 100 chemotactic steps is illustrated in Figure 27.

## Conclusions

In this paper, a new computational model, termed LCM-QS (lifecycle model with quorum sensing mechanism) is proposed to simulate emergent behaviour of bacterial quorum sensing. The communication mechanism is the most important factor to indicate a swarm intelligence system. The artificial bacteria are endowed with communication ability by using the principle of swarm intelligence. Additionally, reproduction, elimination and migration are all viewed as optimization strategies to build the LCM-QS model. To illustrate the performance of the proposed model, four types of communication schemes between individuals or groups are studied by adapting a 3-D artificial environment with nutrient-noxious distribution. The results show that by using quorum sensing mechanism artificial bacteria are able to response quickly to the complex environment and can find the global optimum in a short time.

The primary goal of this paper concentrates on developing a novel individual-based modelling approach to simulate the quorum sensing mechanism among bacterial colonies. Meanwhile, the LCM-QS model is expected to give an inspiration to present a new swarm intelligence optimization algorithm. However, little consideration is given to other factors such as varying population, dynamic environment. Therefore, in our future work, these issues will be focused on and some real-world applications will be considered, such as the design of new evolutionary neural networks [27-30].

## Competing interests

The authors declare that they have no competing interests.

## Authors' contributions

NB. defined the research question and initiated the ideas. NB, WH designed and conducted simulation experiments. NB, WH, DQQ drafted the manuscript. LL defined the final manuscript. All authors contributed to and approved the final manuscript.

**Figure 21:**
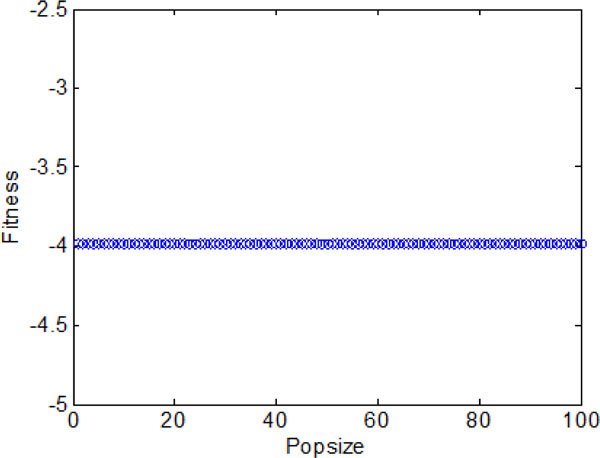
**The final optimal function values of each bacterium with both individual and group information exchange**.

## Declarations

This article has been published as part of *BMC Bioinformatics *Volume 14 Supplement 8, 2013: Proceedings of the 2012 International Conference on Intelligent Computing (ICIC 2012). The full contents of the supplement are available online at http://www.biomedcentral.com/bmcbioinformatics/supplements/14/S8.
